# Complete mitochondrial genome of Cominate sea catfish *Occidentarius platypogon* (Siluriformes: Ariidae)

**DOI:** 10.1080/23802359.2017.1334516

**Published:** 2017-06-01

**Authors:** Raúl Llera-Herrera, Jorge S. Ramírez-Pérez, Nancy C. Saavedra-Sotelo

**Affiliations:** a Unidad Acuicultura y Manejo Ambiental, CONACYT-Centro de Investigación en Alimentación y Desarrollo A. C., Mazatlán, Sinaloa, Mexico;; b Facultad de Ciencias del Mar, Universidad Autónoma de Sinaloa, Mazatlán, Mexico;; c Facultad de Ciencias del Mar, CONACYT-Universidad Autónoma de Sinaloa, Mazatlán, Mexico

**Keywords:** Cominate sea catfish, *Occidentarius platypogon*, mitogenome, *de novo* assembly

## Abstract

The mitochondrion genome of *Occidentarius platypogon* was assembled from Illumina short reads, and consisted of 16,714 base pairs, with 13 protein-coding, two ribosomal RNAs (rRNAs), and 22 transfer RNA (tRNA) genes. Base composition is 30.7% A, 26.4% T, 28.5% C, and 14.4% G, and 42.9% GC content. Two start codon (ATG and GTG) and seven stop codon (TAA, ACT, CCT, TTA, CAT, AAT, and TAG) patterns were found in protein-coding genes. Control region presented the highest A + T (64%) and lowest G + C content (35.7%) among all mitochondrial regions.

The cominate catfish, *Occidentarius platypogon,* belongs to a monotypic genus which include only this species, and its distribution is limited to the Eastern Pacific from Mexico to Peru (Betancur-R et al. 2007). This species is of relevant interest for artisanal fishing along the Sinaloa coast (northwest of Mexico), and is also incidentally caught by industrial and artisanal shrimp fisheries (Amezcua et al. 2006; Madrid-Vera et al. 2007; Amezcua & Muro-Torres 2012). In spite of abundance and economic importance of this species, there are few studies that describe their biology, ecology and genetics. In Mazatlán coast, *O. platypogon* present a synchronic gonad development and a spawning season from May to August, with males incubating the eggs in the oral cavity (Amezcua & Muro-Torres 2012).

To determine the complete mitochondrial genome of *O. platypogon*, we collected a specimen captured in Sinaloa, Mexico (23°28′32.5″N–106°37′28.2″W). DNA was extracted from fresh muscle tissue using the Wizard® Genomic DNA Purification kit (Promega, Madison, WI). A genomic DNA library was constructed with the Kapa gDNA library preparation kit (Kapa Biosystems, Wilmington, MA) using multiplex index, and the library was then sequenced alongside other barcoded libraries using a single lane (2 × 125 paired-end reads) in a MiSeq platform (Illumina, San Diego, CA). After demultiplexing, reads were pre-processed using Trimmomatic 0.33 (Bolger et al. 2014) for trim low-quality ends (Q score <20), residual adapters and remove reads shorter than 100 bases. All reads were analysed for quality control (QC) with FastQC v0.10.1 (Babraham Institute, Cambridge, UK) (Andrews 2011) before and after quality trimming. 21’391,977 pair-end reads were finally obtained.

Using the complete mitochondrion genome of *Netuma thalassina* (GenBank accession number: KU986659.1) as a reference, the complete genome was obtained by multi-step assembly (7 iterations) using the MITObim ver.1.7 (Hahn et al. 2013). The mitogenome of *O. platypogon* (GenBank accession number KY930717) has a length of 16,714 bp and a base composition of A 30.7%, T 26.4%, C 28.5%, and G 14.4%, and the GC content of 42.9%. The complete mitochondrial genome contains all typical genes found in most vertebrate mitogenomes: 13 protein-coding genes, 22 transference RNA genes, two ribosomal RNAs, and one control region or d-loop (Figure 1). Protein coding genes initiate by the typical ATG codon, except for the *COX1* gene, which presented GTG as start codon. Almost all genes presented TAA or ACT as stop codon, the rest used a different one (Table 1). *D-loop* region was 1123 bp, presenting the highest A + T content of 64.3% among all mitochondrial regions. No evidence of SNP variation over a frequency of 0.05 was found.

**Figure 1.**
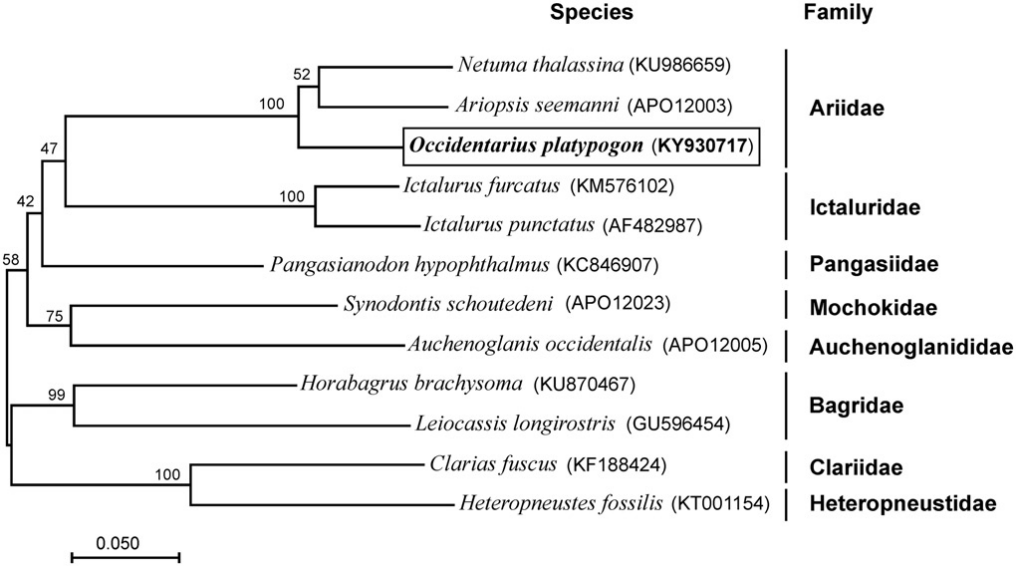
Maximum-likelihood (ML) phylogenetic tree of *Occidentarius platypogon* and the other 11 species of 8 families using *Clarias fuscus* and *Heteropneustes fossilis* as an outgroup. Number above each node indicates the ML bootstrap support values. In parenthesis the access numbers from NCBI database.

**Table 1.** Annotation of the complete mitochondrial genome of *Occidentarius platypogon*.

Gene name	Location (bp)	Length (bp)	Start codon	Stop codon
*tRNA-Phe*	1–70	70		
*12SrRNA*	71–1025	955		
*tRNA-Val*	1026–1097	72		
*16SrRNA*	1098–2769	1672		
*tRNA-Leu*	2770–2845	76		
*NAD1*	2846–3820	975	ATG	TAA
*tRNA-Ile*	3821–3892	72		
*tRNA-Gln*	3893–3963	71		
*tRNA-Met*	3964–4033	70		
*NAD2*	4034–5078	1045	ATG	ACT
*tRNA-Trp*	5079–5151	73		
*tRNA-Ala*	5152–5220	69		
*tRNA-Asn*	5221–5293	73		
*tRNA-Cys*	5294–5360	67		
*tRNA-Tyr*	5361–5430	70		
*COX1*	5431–6981	1551	GTG	TAA
*tRNA-Ser*	6982–7052	71		
*tRNA-Asp*	7053–7121	69		
*COX2*	7122–7812	691	ATG	CCT
*tRNA-Lys*	7813–7886	74		
*ATP8*	7887–8054	168	ATG	TAA
*ATP6*	8055–8737	683	ATG	TTA
*COX3*	8738–9521	784	ATG	CAT
*tRNA-Gly*	9522–9594	73		
*NAD3*	9595–9943	349	ATG	AAT
*tRNA-Arg*	9944–10,014	71		
*NAD4L*	10,015–10,311	297	ATG	TAA
*NAD4*	10,312–11,692	1381	ATG	ACT
*tRNA-His*	11,693–11,762	70		
*tRNA-Ser*	11,763–11,829	67		
*tRNA-Leu*	11,830–11,902	73		
*NAD5*	11,903–13,729	1827	ATG	TAA
*NAD6*	13,730–14,242	513	ATG	TAG
*tRNA-Glu*	14,243–14,311	69		
*CYTB*	14,312–15,449	1138	ATG	ACT
*tRNA-Thr*	15,450–15,521	72		
*tRNA-Pro*	15,522–15,591	70		
*D-loop*	15,592–16,714	1123		

We validated the phylogenetic position of *O. platypogon* with a maximum-likelihood tree (500 boostrap replicates) of complete mtDNA from the other 11 species using MEGA6 (Tamura et al. 2013). The phylogenetic position of *O. platypogon* was closely clustered with two species of Ariidae family (Figure 1). The newly determined mitogenome will help to understand the evolution of Ariidae family.
